# Suppressors of *lapC* Mutation Identify New Regulators of LpxC, Which Mediates the First Committed Step in Lipopolysaccharide Biosynthesis

**DOI:** 10.3390/ijms242015174

**Published:** 2023-10-14

**Authors:** Akshay Maniyeri, Alicja Wieczorek, Aravind Ayyolath, Weronika Sugalska, Gracjana Klein, Satish Raina

**Affiliations:** Laboratory of Bacterial Genetics, Gdansk University of Technology, 80-233 Gdansk, Poland; akshay.m.s@pg.edu.pl (A.M.); alicja.wieczorek@pg.edu.pl (A.W.); s192477@student.pg.edu.pl (A.A.); s180651@student.pg.edu.pl (W.S.)

**Keywords:** lipopolysaccharide (LPS), LPS assembly proteins LapB, LapC and LapD, MarA, LpxC, acyltransferases LpxL and LpxM

## Abstract

Gram-negative bacteria, such as *Escherichia coli*, are characterized by an asymmetric outer membrane (OM) with lipopolysaccharide (LPS) located in the outer leaflet and phospholipids facing the inner leaflet. *E*. *coli* recruits LPS assembly proteins LapB, LapC and LapD in concert with FtsH protease to ensure a balanced biosynthesis of LPS and phospholipids. We recently reported that bacteria either lacking the periplasmic domain of the essential LapC protein (*lapC190*) or in the absence of LapD exhibit an elevated degradation of LpxC, which catalyzes the first committed step in LPS biosynthesis. To further understand the functions of LapC and LapD in regulating LPS biosynthesis, we show that the overproduction of the intact LapD suppresses the temperature sensitivity (Ts) of *lapC190*, but not when either its N-terminal transmembrane anchor or specific conserved amino acids in the C-terminal domain are mutated. Moreover, overexpression of *srrA, marA, yceJ* and *yfgM* genes can rescue the Ts phenotype of *lapC190* bacteria by restoring LpxC amounts. We further show that MarA-mediated suppression requires the expression of *mla* genes, whose products participate in the maintenance of OM asymmetry, and the SrrA-mediated suppression requires the presence of cardiolipin synthase A.

## 1. Introduction

**Figure 1:**
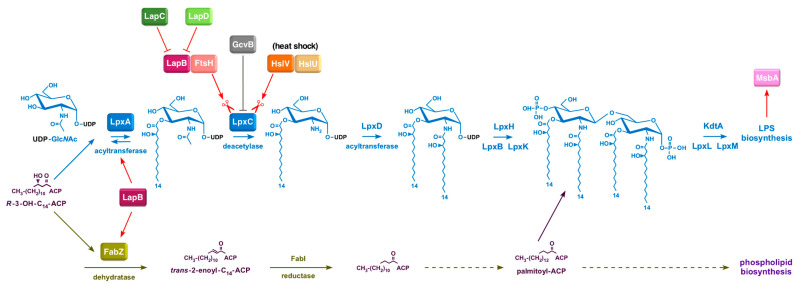
Schematic drawing depicting various players that regulate the first committed step in LPS biosynthesis catalyzed by LpxC and maintain a balance between LPS and phospholipids. Shown is the utilization of the common metabolic precursor (*R*)-3-hydroxymyristate by LpxA and by FabZ in LPS and phospholipid biosynthesis, respectively. LpxC amounts are regulated by its turnover by the FtsH-LapB complex and at high temperature by HslVU protease. LapC and LapD act as antagonist of LapB to regulate LPS biosynthesis as per its demand. Also shown is LapB interaction with LpxA and FabZ, thus acting as a central hub. LpxC amounts can also be fine-tuned by GcvB sRNA.

The most distinguishing feature of Gram-negative bacteria, such as *Escherichia coli*, is the presence of an asymmetric outer membrane (OM) [1]. This asymmetry is due to the characteristic location of lipopolysaccharide (LPS) in the outer leaflet of the cell envelope, with phospholipids facing its inner leaflet [1,2]. The maintenance of this OM asymmetry is essential to impart a permeability barrier, which prevents the entry of bulky toxic molecules, such as antibiotics and bile salts, inside the cells. Thus, with few exceptions, LPS is essential for bacterial viability and is one of the major virulence factors in pathogenic Gram-negative bacteria. LPS is a complex glycolipid and, in general, they share a common architecture and can be, for convenience, divided into three parts. The most conserved component is the hydrophobic membrane-anchored lipid A part, which constitutes the endotoxin principal. To the lipid A part is attached a core oligosaccharide, to which an oligosaccharide of variable length called the *O*-antigen is attached in bacteria with smooth LPS [3]. The biosynthesis of LPS at the genetic and biochemical levels is well established, although how LPS amounts are regulated and its final assembly in the OM is not well understood. This is particularly important because bacteria must maintain a strict balance of 1:0.15 between phospholipids and LPS, the two essential components of the cell envelope [4]. In *E*. *coli*, this is achieved by regulating the first committed step in LPS biosynthesis, catalyzed by the essential enzyme LpxC, because LPS and phospholipids use the same (*R*)-3-hydroxymyristate as the common metabolic precursor [5]. However, the regulation of LpxC as we understand now turns out to be more complex than it was thought a few years ago, as it responds to multiple physiological cues and a regulated turnover by FtsH and HslVU proteases (Figure 1) [5,6].

In *E*. *coli*, the biosynthesis and transport of LPS require products of more than thirty genes and many of them are essential for bacterial viability [2]. Briefly, LPS biosynthesis occurs on the inner leaflet of the inner membrane and begins with the acylation of UDP-GlcNAc, catalyzed by LpxA. In *E*. *coli*, LpxA is selective for *β*-hydroxymyristoyl acyl carrier protein [7]. This is followed by deacetylation of UDP-3-*O*-(acyl)-GlcNAc by the Zn^2+^-dependent LpxC enzyme [8]. As the equilibrium constant for LpxA-mediated acylation is unfavorable, LpxC-catalyzed deacetylation becomes the first committed step in lipid A biosynthesis [9]. Following the deacetylation step, a second *β*-hydroxymyristoyl chain is incorporated by *E. coli* LpxD, leading to the synthesis of UDP-2,3-diacyl-GlcN [10]. Three additional enzymes, LpxH, LpxB and LpxK, act successively to generate the intermediate lipid IV_A_ [11]. This intermediate serves as an acceptor for the incorporation of two 3-deoxy-α-D-*manno*-oct-2-ulosonic acid (Kdo) residues by WaaA, with CMP-Kdo as the donor, generating Kdo_2_-lipid IV_A_ [12]. In *E*. *coli*, up to the step of Kdo_2_-lipid IV_A_ biosynthesis, all enzymes are essential for bacterial viability, although strains synthesizing only a lipid IV_A_ precursor or Kdo_2_-lipid IV_A_ can be constructed [13]. The incorporation of Kdo residues serves as a key intermediate to generate hexaacylated lipid A via sequential acylation by lauroyl (LpxL) and myristoyl (LpxM) transferases and further incorporation of sugar residues using specific glycosyltransferases to produce a core oligosaccharide linked to the lipid A part [13,14]. Strains synthesizing tetraacylated lipid A can only grow on minimal medium and exhibit an elevated cell envelope stress response [13,14]. Interestingly, suppressors mapping to the *msbA* gene encoding LPS flippase can allow the growth of strains devoid of LpxL, LpxM and LpxP on rich medium and at elevated temperatures. These observations are in agreement with the known higher selectivity exerted by MsbA for hexaacylated lipid A as compared to the less favored underacylated species. Individually, all three late acyltransferases are nonessential, although Δ*lpxL* exhibits a temperature-sensitive growth phenotype. However, LpxL and LpxM become essential when cardiolipin synthase A, encoded by the *clsA* gene, is absent, suggesting that MsbA and ClsA could cooperate in LPS trafficking, although the precise mechanism of their interaction remains unknown [15,16]. Thus, besides the regulatory control exerted by the regulation of LpxC amounts, the preferential selection of hexaacylated lipid A species by MsbA constitutes early checkpoints in controlling the proper amounts of LPS and its translocation across the inner membrane [17].

Initial studies on the regulation of LpxC deacetylase revealed that its levels are controlled in a 20-fold range in relation to the lipid A content without any increase in the corresponding mRNA levels of the encoded gene [18]. Subsequently, it was shown that LpxC is a substrate of FtsH protease and this proteolytic activity might be affected by alterations in acyl-ACP pools [5]. Such a model of FtsH-mediated proteolysis of LpxC could, in turn, provide a balance between biosynthesis of phospholipids and lipid A since both derive their fatty acyl chains from the same (*R*)-3-hydroxyacyl-ACP pool (Figure 1) [5]. In an important breakthrough, a new essential inner membrane protein was identified and shown to co-purify with FtsH and several proteins involved in either LPS and phospholipid biosynthesis or LPS translocation, and hence designated the LPS assembly protein LapB [19]. The absence of LapB could be tolerated either in the presence of a hyperactive *fabZ* variant that allowed an *ftsH* deletion or when the biosynthesis of LPS was reduced [19,20]. The co-purification of LapB with FtsH, FabZ, WaaC and Lpt proteins led to the proposal that LapB could act as a hub for LPS assembly in the IM and further cooperate with FtsH to regulate LpxC turnover [19]. More recent studies have validated these earlier observations and indeed, LapB was found to interact with LpxA, LpxC, LpxD and FabZ [21]. Similarly, recent structural and biochemical studies have shown that LpxC degradation by FtsH requires LapB [22]. However, how such LpxC degradation by FtsH-LapB is adjusted to the cellular demand for LPS remained unknown till the more recent discovery of LapC [2]. We and other independent research groups have shown that LapC acts as an antagonist of the proteolytic pathway of LpxC mediated by FtsH-LapB [6,22,23,24,25,26]. Our results were further supported by an examination of the properties of strains with chromosomal mutations that cause truncations in the periplasmic domain of LapC [6]. Such mutant bacteria exhibited a temperature-sensitive (Ts) phenotype, hypersensitivity to the LpxC inhibitor CHIR090, hyperdegradation of LpxC and consequently reduced levels of LPS (Figure 1). Thus, not only our group, but parallel independent studies, identified extragenic suppressors of *lapC* mutant bacteria mapping to the *lapA*/*B* operon, *lpxC* and *ftsH* genes [6,23,24,26,27]. Furthermore, an additional partner, LapD, which also co-purifies with LapB, was identified [28]. Δ*lapD* bacteria were found to exhibit a Ts phenotype and membrane permeability defects with a synthetic lethality in the absence of either cardiolipin synthase A, or when LpxL or LpxM acyltransferase was absent or when bacteria synthesize LPS composed of only Kdo_2_-lipid A due to the absence of WaaC heptosyltransferase [28,29]. Moreover, extragenic suppressors that overcome defects of *lapC* mutant bacteria were also found to rescue the Ts phenotype or vancomycin sensitivity of Δ*lapD* bacteria [28].

**Figure 2:**
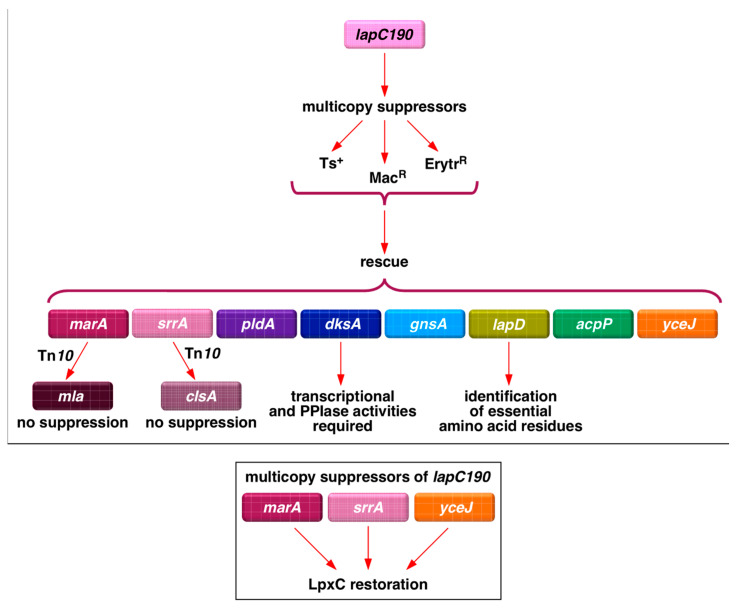
Schematic depiction of various approaches that identify new regulators of LpxC. Multicopy suppressors of sensitivity to high temperature, growth on either MacConkey agar or erythromycin of *lapC190* identified several genes, some of which act by increasing LpxC and LPS amounts (bottom panel). Transposon mutagenesis showed that while as MarA-mediated suppression requires the functional presence of Mla system, SrrA-dependent suppression needs the presence of cardiolipin synthase A.

However, how LapB, LapC and LapD sense LPS biosynthetic demand and whether there are additional factors that either inhibit or enhance LpxC degradation remain unknown. Adding to this complexity, LpxC can be degraded in the absence of LapB by HslVU, and levels of LpxC can also be regulated by regulatory small RNAs, although the precise mechanism remains unknown (Figure 1) [6]. LPS synthesis can also be stimulated by additional signals that may involve systems that respond to lipid asymmetry, including the activation of outer membrane phospholipase PldA, the Mla system that facilitates retrograde lipid trafficking, and PagP palmitoyltransferase [30]. LpxC stability can further respond to changes in levels of acyl-CoA, ppGpp and alterations in the levels of saturated fatty acids as compared to unsaturated [17,31]. Thus, the regulation of LpxC and, in turn, that of LPS involves several factors. Hence, in this study, we identified additional genes whose products might regulate LpxC or LPS levels by selecting for suppressors whose overexpression can overcome the Ts phenotype of *lapC* mutant bacteria (Figure 2). We show that overexpression of the *lapD* gene and some other potential new regulatory factors (MarA, SrrA, DksA and YceJ) can suppress the Ts phenotype of *lapC190* mutant bacteria and, among these, some act by increasing the amount of LpxC. MarA is a well-characterized transcription factor that regulates the expression of several genes involved in regulating antibiotic resistance [32]. Using transposon mutagenesis, gene(s) whose products are required for MarA- and SrrA-mediated suppression were identified to gain a better understanding of that factors that limit bacterial growth when LapC is dysfunctional and additional players that regulate LpxC amounts (Figure 2).

## 2. Results

### 2.1. Genes Whose Overexpression Overcomes the Temperature Sensitivity (Ts) of lapC Mutant Bacteria with a Truncation of LapC Periplasmic Domain Identify New Players That Can Regulate LpxC Amounts

**Figure 3:**
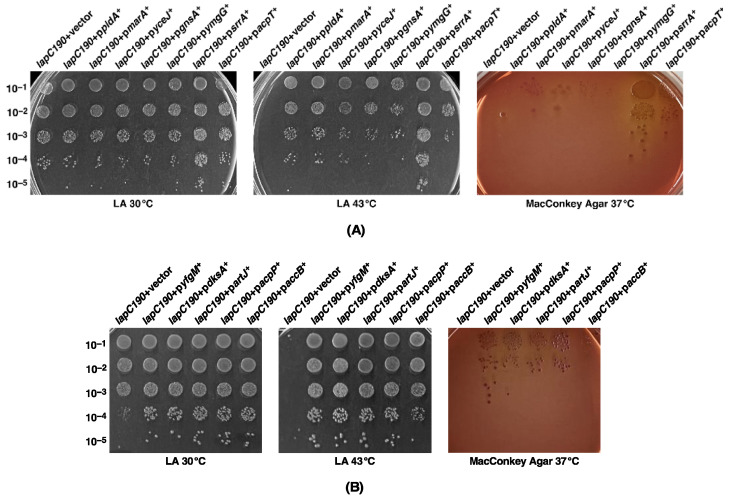
Overexpression of specific genes, including *marA, srrA* and *acpP* genes, restore the growth of *lap190* bacteria at 43 °C. Overexpression of some of them, such as *srrA* (**A**) and *yfgM* (**B**), also restore growth on MacConkey agar. Growth of isogenic cultures of *lap190* bacteria transformed with either plasmid DNA of the vector alone or when the specific multicopy suppressing gene is present on the plasmid was quantified by spot dilution on LA 30 °C and 43 °C. For the restoration of growth on MacConkey agar, plates were incubated at 37 °C (**A**,**B**). The relevant genotype and temperature of incubation are indicated.

**Figure 4:**
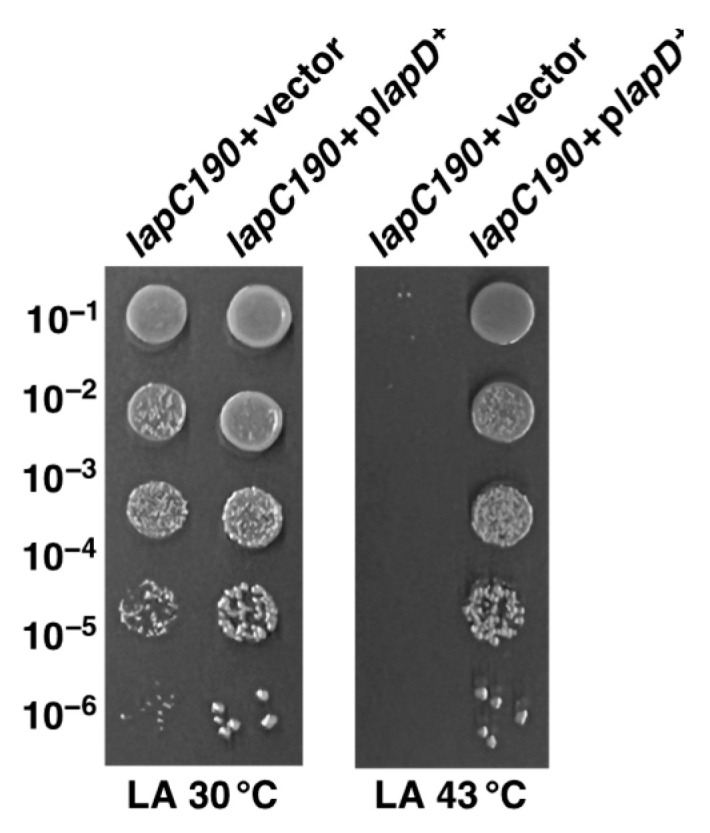
Overexpression of the *lapD* gene can restore the growth of *lap190* bacteria at 43 °C. Growth of isogenic cultures of *lap190* bacteria transformed with either plasmid DNA of the vector alone or when the *lapD* gene is present on the plasmid was quantified by spot dilution on LA 30 °C and 43 °C. The relevant genotype and temperature of incubation are indicated.

We previously reported the isolation of mutations in the essential gene encoding the LapC protein. Such mutations cause truncation in the periplasmic domain of LapC and confer a Ts phenotype, permeability defects and a reduced amount of LPS due to the hyperdegradation of LpxC [6]. Some of these phenotypic defects could be suppressed by chromosomal single-copy mutations mapping to *lpxC*, *ftsH* and the *lapA*/*B* operon [6]. This suppression was attributed to the stabilization of LpxC. However, since several factors can contribute towards the regulation of LpxC, here we asked if the overexpression of any gene can also suppress growth defects of *lapC* mutant bacteria. Thus, we used *lapC190* mutant bacteria, which lack the periplasmic domain of LapC on the chromosome, to isolate multicopy suppressors, using a complete genomic library. To achieve this goal, plasmid DNA pools from the ASKA collection of single ORFs expressed from the tightly regulated IPTG-inducible P_T5_-*lac* promoter in the vector pCA24N [33] were used to transform competent cells of *lapC190* mutant bacteria. Transformants were selected for the restoration of growth on LA medium supplemented with 75 μM IPTG at 43.5 °C, a condition that is non-permissive for *lapC190* mutant bacteria. This concentration of inducer of gene expression has been optimized as described earlier [19]. Cultures from identified Ts^+^ colonies were used to extract plasmid DNA to retransform *lapC190* bacteria. Plasmids that bred true were retained and their DNA was sequenced, which identified 13 unique genes, whose mild overexpression can rescue the Ts phenotype of *lapC190* mutant bacteria (Table 1, Figure 3). Based on their known or predicted function, these genes can be grouped into various classes: those with a role in LPS, phospholipid/fatty acid biosynthesis or sensing (*lapD*, *acpP, acpT*, *accB*, *pldA*) (Figure 3 and Figure 4), transcription factors (*marA*, *dksA*, *srrA*), an IM-anchored heat shock protein-encoding gene with an unknown function (*yceJ*) and others whose products could either titrate the FtsH protease responsible for LpxC degradation (*yfgM*) or modulate saturated versus unsaturated fatty acid abundance (*gnsA*) or related with the stress response (*ymgG*), whose product is the part of toxin–antitoxin system. 

Next, we examined the suppression of permeability and growth defects. For such studies, we investigated the degree of restoration of bacterial growth/viability. Thus, spot dilution assays were performed on LA medium and on MacConkey agar supplemented with 75 μM IPTG or when supplemented with 15 μg/mL of erythromycin. Data are presented for growth on MacConkey agar as a similar pattern was observed with resistance to erythromycin. These experiments revealed that although most of the multicopy suppressors restored bacterial growth on LA agar at 43.5 °C, the permeability defect was suppressed to a variable extent (Figure 3). Even on LA medium, the colony-forming ability and the colony size indicated the degree of suppression of Ts phenotype was variable. For example, the overexpression of the *acpT* gene conferred only a partial suppression (Figure 3). Thus, on MacConkey agar, only overexpression of *srrA* and *yfgM* genes restored growth to a near-wild-type level. On this medium, which reflects a permeability phenotype, a modest suppression is also conferred by the overexpression of *dksA*, *acpP* and *acpT* genes (Figure 3). Noteworthy of these suppressor-encoding genes is the isolation of a set of genes (*srrA*, *dksA*, *acpP*, *yfgM*, *ymgG, artJ* and *accB*) whose overexpression was earlier shown to suppress the Ts phenotype of Δ*lapD* bacteria [28]. Such results suggest that some common defects in bacteria that are either lacking the periplasmic domain of LapC or when LapD is absent, can be rescued by overexpression of this set of genes (Table 1). However, the identification of *marA* and *pldA* genes is interesting, and their overexpression only suppresses the Ts phenotype of *lapC190* bacteria but not of Δ*lapD* (Table 1). MarA (multiple antibiotic resistance) is a well-characterized transcriptional regulator, known to regulate the expression of several genes whose products are required for antibiotic resistance, oxidative stress and heavy metals tolerance [32,34,35]. Similarly, overexpression of the *acpT* gene only suppresses the Ts phenotype of *lapC190* bacteria but not of the Δ*lapD* bacteria. Thus, although some suppressors identified in this work are common to Δ*lapD* and *lapC190*, the overexpression of some genes such as *marA*, *pldA* and *acpT* is unique for overcoming the Ts phenotype of only *lapC190* bacteria, suggesting a partial overlap in the function of LapC and LapD and not all interacting partners could be the same. These conclusions draw further support from results showing that while in multicopy the *lapD* gene can suppress the Ts phenotype of *lapC190* bacteria (Figure 4), but in converse, overexpression of the *lapC* gene does not rescue the Ts phenotype of Δ*lapD* bacteria.

### 2.2. Overexpression of srrA, marA, yceJ and yfgM Restore LpxC Amounts in lapC Mutant Bacteria

**Figure 5:**
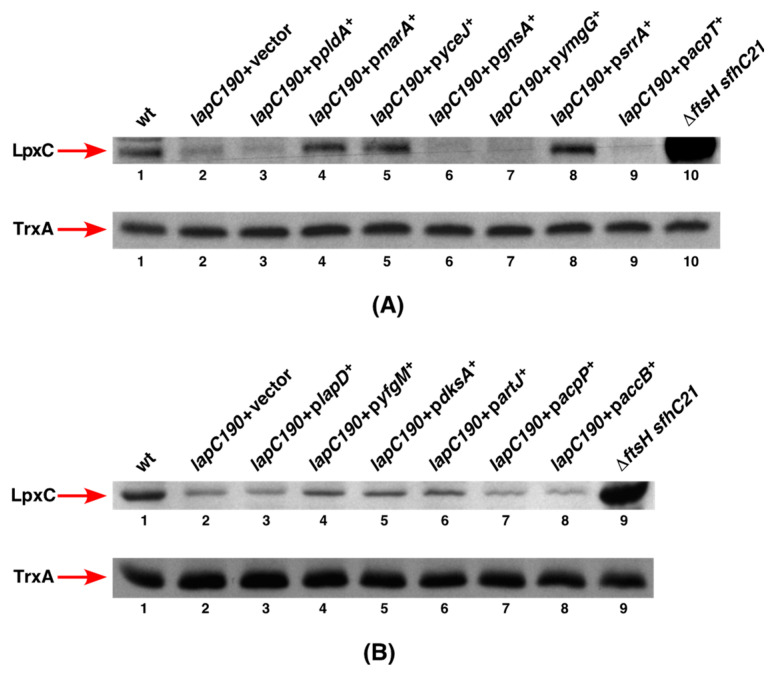
Restoration of LpxC amounts to different extents upon the overexpression of various multicopy suppressor encoding genes in *lap190* bacteria. Immunoblots of whole cell lysates obtained from isogenic strains grown at 30 °C, followed by the addition of 75 μM IPTG and a temperature shift for 2 h at 43 °C (**A**,**B**). For immunoblotting, LpxC-specific antibodies were used and the relevant genotype is indicated. An equivalent amount of total proteins was resolved by an SDS-PAGE prior to immunoblotting. As controls, lane 2 in panel (**A**) and panel (**B**) samples from *lapC190* bacteria are loaded, which have reduced LpxC amounts. Additional control involves samples from Δ*ftsH sfhC21* which expectedly reveal higher amounts of LpxC due to its stabilization lane 10 (panel (**A**)) and lane 9 (panel (**B**)). As loading control, the same samples were probed with anti-TrxA antibodies.

To address the molecular basis of the suppression of growth defects at elevated temperatures by overexpression of the above-mentioned multicopy suppressor-encoding genes, experiments were undertaken to examine the amount of LpxC upon their mild induction. The rationale for these studies is to verify if the major defect reflected by the reduction in LpxC amounts associated with the absence of the periplasmic domain of LapC can be overcome by overexpression of any of these genes. Thus, total cell extracts were obtained from the wild type and panels of isogenic Δ*lap190* derivatives either with an empty vector or when the overexpressing plasmid was present under conditions described in the legend to Figure 5. The equivalent amount of proteins was resolved on a 12% SDS-PAGE, and LpxC was detected by immunoblotting with LpxC-specific antibodies. The estimation of relative amounts of LpxC in such experiments prominently shows that induction of expression of *marA*, *yceJ* and *srrA* restore LpxC to near wild-type levels (Figure 5A, lanes 4, 5 and 8). We applied the same amounts of total proteins from the Δ*ftsH sfhC21* strain since, in the absence of FtsH, LpxC amounts are increased to serve as a marker for LpxC and as a control. In parallel, the same samples were immunoblotted with anti-TrxA antibodies serving as an additional loading control. Among the remaining genes, whose overexpression also suppresses the Ts phenotype of *lapC190* bacteria, a modest increase in LpxC abundance can be observed in strains with induced expression of either *yfgM* or *dksA* or *artJ* genes in the *lapC190* background (Figure 5B, lanes 4, 5 and 6). These results suggest that the induction of *marA*, *yceJ* and *srrA* genes and, to a certain extent, of *yfgM*, *dksA* and *artJ* can suppress the growth defect of *lapC190* bacteria by restoring LpxC amounts. Surprisingly, the overexpression of *pldA*, *gnsA*, *ymgG* and *acpT* genes (Figure 5A) as well as *lapD*, *acpP* and *accB* (Figure 5B) did not cause any noticeable increase in LpxC amounts and remained similar to when extracts from *lapC190* with the vector alone were applied. It is possible that in the case of expression of such multicopy-encoding suppressor genes, a mild induction of their expression may not be sufficient to significantly increase LpxC amounts, although Ts of *lapC190* bacteria can be suppressed. Overall, we can conclude that a mild overproduction of SrrA, MarA, YceJ and YfgM suppress the Ts phenotype and also lead to the restoration of LpxC amounts, explaining the molecular basis of their contribution in the regulation of LpxC. 

### 2.3. Overexpression of srrA, marA, yceJ and dksA also Restore LPS Amounts in lapC190 Bacteria

We previously showed that *lapC190* bacteria have reduced amounts of LPS due to the enhanced degradation of LpxC as compared to the wild type. Thus, to address the mechanism of suppression by overexpression of specific genes that provide a robust restoration of growth at elevated temperatures, we estimated levels of LPS from total cell extracts. For such experiments, we measured levels of LPS from cellular extracts obtained from exponentially grown cultures of the isogenic wild type, its *lapC190* derivative with the vector alone and *lapC190* bacteria carrying plasmid that expresses a specific suppressor encoding gene under an inducible P_T5_-*lac* promoter. Bacterial cultures were grown under permissive growth conditions at 30 °C in LB medium, the gene expression was induced with the addition of 75 μM IPTG at OD_595_ 0.1 and samples obtained under the same conditions when LpxC amounts were measured as described in Figure 5. Total cell extracts were also applied from isogenic Δ*ftsH sfhC21* derivative as a control. Equivalent amount of whole cell lysates was treated with Proteinase K. Samples were resolved on an SDS-Tricine gel and LPS was transferred by Western blotting. Amounts of LPS were revealed using an LPS-specific WN1 222-5 monoclonal antibody as described in the Methods section. 

**Figure 6:**
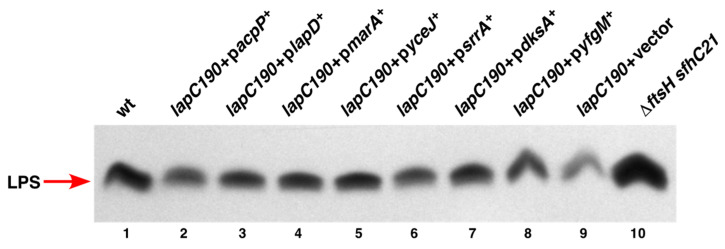
Restoration of LPS amounts by overexpression of *marA*, *yceJ*, *srrA* and *dksA* to different extents in *lap190* bacteria. Immunoblots of whole cell lysates obtained from isogenic strains grown at 30 °C, following a temperature shift for 2 h at 43 °C. Expression of the specific gene was induced by the addition of IPTG. An equivalent portion of whole cell lysates were applied and samples were resolved on a 15.5% SDS-Tricine gel and LPS was transferred by Western blotting. Amounts of LPS were revealed using a LPS-specific WN1 222-5 monoclonal antibody. For immunoblotting, LpxC-specific antibodies were used and the relevant genotype is indicated. An equivalent amount of total proteins was resolved by an SDS-PAGE prior to immunoblotting using chemiluminescence detection kit.

The estimation of relative amounts of LPS in such experiments show that *lapC190* bacteria have highly diminished amounts of LPS (Figure 6 lane 9) as compared to the wild type and the Δ*ftsH sfhC21* derivative contains elevated amounts of LPS. Most importantly, results from such immunoblots reveal that extracts from the *lapC190* derivative overexpressing either *marA*, or *yceJ*, or *srrA* or the *dksA* gene show the restoration of LPS amounts to near wild-type levels explaining the mechanism of their suppression (Figure 6). As with the estimation of LpxC amounts, overexpression of the *acpP* gene did not cause any increase in the amounts of LPS and remained low as observed with *lapC190* bacteria. Similarly, overexpression of the *lapD* gene only marginally enhances LPS amounts. These results allow us to conclude that some of the multicopy suppressors such as *marA*, *dksA*, *srrA* and *yceJ* contribute via increasing LpxC and LPS amounts, providing a reasonable explanation for their identification. However, the mode of restoration of growth of *lapC* mutant bacteria by an overexpression of the *acpP* gene could involve the LpxC independent mode.

### 2.4. srrA, marA, lapD and pldA Are Indispensable in the lapC190 Mutant Background

To further understand the mechanism of suppression by various genes whose increased expression mitigates the Ts phenotype of *lapC190* bacteria, a systematic series of bacteriophage-mediated transduction was executed. This was to ascertain if there are any genetic interactions and if any of such genes are essential for the growth and viability of *lapC190* bacteria. We focused on those genes, whose overexpression also restores LpxC levels. Thus, null alleles of such candidates were introduced in *lapC190* bacteria. In parallel, converse reverse transductions were also carried out by attempting to bring in a *lapC190* mutation in deletion derivatives of genes whose overexpression results in the restoration of growth. Such transductions revealed that the presence of SrrA, MarA, LapD and PldA are absolutely required for the viability of *lapC190* bacteria even at 30 °C but not in the wild-type parental strain (Table 2). Thus, no viable transductional Δ*srrA lapC190* and Δ*lapD lapC190* combinations could be obtained. However, when the wild-type copy of the *srrA* or *lapD* gene was provided on a plasmid, viable chromosomal Δ*srrA lapC190* and Δ*lapD lapC190* mutational combinations were obtained at the same frequency when the wild-type parental strain was used as a recipient to bring in either *srrA* or *lapD* null mutations (Table 2 and Table 3). In contrast, the Δ*dksA lapC190* mutational combination was found to be viable up to 37 °C, although the colony size was heterogeneous. A deletion of either the *marA* gene or the *pldA* gene could be introduced in *lapC190* bacteria, albeit at a highly reduced frequency, and the majority of such transductants were found to be not viable (Table 2). These genetic interactions allow us to conclude that SrrA, MarA, LapD and PldA are essential for the viability of strains lacking the chromosome of the DNA sequence encoding the periplasmic domain of LapC. 

### 2.5. Lauroyl and Myristoyl Transferases Are Required for the Viability of lapC190 Mutant Bacteria

Next, we examined if acyltransferases that mediate the transfer of lauroyl (LpxL) and myristoyl (LpxM) acyl chains to Kdo_2_-lipid IV_A_ are essential for the growth of *lapC190* mutant bacteria. Bacteriophage P1-mediated transfer of *lapC190* or an introduction of Δ*lpxL* or Δ*lpxM* revealed that Δ*lpxL lapC190* and Δ*lpxM lapC190* mutational combinations are only possible in the ectopic presence of covering plasmid containing the wild-type gene (Table 2 and Table 3). However, at a very low frequency, viable suppressors could be obtained after an incubation of more than 48 h when Δ*lpxL lapC190* and Δ*lpxM lapC190* transductants were plated. Further characterization of two suppressors of each such mutational combinations identified a single amino acid exchange, L412P, in the *msbA* gene. Interestingly, the same *msbA* suppressor mutation was found to overcome the lethality of Δ(*lpxL lapD*) and Δ(*lpxM lapD*) combinations [28]. Thus, we can conclude that truncation of the *lapC* gene, resulting in the expression of only the transmembrane anchor, requires complete acylation of the lipid A. Since underacylated LPS (lack of LpxL or LpxM) is poorly translocated by MsbA as compared to hexaacylated lipid A, this could lead to a further reduction in the amount of LPS in the OM in *lapC190* bacteria and hence the lethality. Taken together, *lapC190* derivatives synthesizing either tetraacylated or pentaacylated lipid A, cannot be constructed unless a suppressor mutation in the *msbA* gene, which encodes lipid A flippase, is present.

### 2.6. MarA-Mediated Suppression Requires the Presence of mla Genes 

As the induction of the *marA* gene expression restored the growth of *lapC190* mutant bacteria at an elevated temperature, we further investigated its possible mechanism of suppression. Thus, strain SR23660 (*lapC190* + p*marA*^+^) served as a host to generate a saturated Tn*10* Kan transposon library at 30 °C. Nearly 50,000 such transposon mutants were screened for the growth on LA medium at 43 °C in the presence of 75 μM IPTG and as a control at 30 °C without the presence of an inducer. This is the strategy that we earlier successfully employed to identify genes whose products are required for the multicopy suppressor phenotype of the *dksA* gene [36]. Thus, we retained those Tn*10* insertion mutants that grew at 30 °C but were unable to propagate at 43 °C, even with the induction of the *marA* gene expression. Such transposon mutations were backcrossed into SR23660 and verified for lack of suppression of *lapC190* mutant bacteria in the presence of the inducible *marA* gene. This resulted in the identification of fifty-four independent Tn*10* mutants. Next, such Tn*10* Kan transposon insertions were introduced into the wild type by bacteriophage P1-mediated transductions to eliminate mutations that conferred a Ts phenotype at 43 °C, even in the wild-type background. Such experiments identified 22 Tn*10* insertions that were found to confer the Ts phenotype in the wild type, while the remaining were specific to the *lapC190* strain. Most of the Ts mutants were found to have the Tn*10* insertion in the *degP* gene, whose product is known to be required for bacterial growth at temperatures above 42 °C [37,38]. This is consistent with a similar spectrum of Ts insertions obtained in an identical strategy described to unravel the requirement of specific genes for DksA-mediated multicopy suppression [36]. The mapping and characterization of the remaining Tn*10* insertions that prevent the multicopy suppression by *marA* identified 12 out of 15 mapping to genes whose products are part of the Mla pathway. To facilitate the mapping of Tn*10* insertions, we used a previously described linked Δ*rpoN* [39] to find how many can cross out the Tn*10* insertion mutation. Such transductions revealed that the majority of Tn*10* insertion mutants had a disruption of the *mla* operon. DNA sequence analysis of the Tn*10* junction from chromosomal DNA of *mla* Tn*10* insertions was obtained by inverse PCR, which identified four insertions each in *mlaC* and *mlaD*, three insertions in *mlaF* and one insertion in the *mlaA* gene. The Mla system is known to be required for the maintenance of outer membrane lipid asymmetry and the inactivation of *mla* genes confers sensitivity to SDS+EDTA [40]. The *mla* operon contains *mlaF*, *mlaE*, *mlaD*, *mlaC* and *mlaB* genes, out of which the *mlaF* gene encodes the ATP-binding protein of an inner membrane complex (MlaFEDB). The Mla pathway functions as part of the retrograde and/or anterograde intermembrane phospholipid trafficking system [40,41]. The Mla system also contains MlaA, whose encoded gene is located outside the *mla* operon. The Mla system can function by removing mislocalized outer leaflet phospholipids and transporting them back to the inner membrane. Thus, the isolation of Tn*10* insertion in genes encoding the Mla pathway that prevent MarA-mediated suppression of *lapC190* bacteria is consistent with LapC function in regulating LPS amounts and a balance with phospholipid amounts. 

Thus, to further support these results, we used previously constructed in-frame deletion derivatives of *mlaC* and *mlaD* [42] and a new construct Δ(*mlaC mlaD*) to verify if such mutations abolish *marA* multicopy suppression. This was required because Tn*10* insertions are expected to be polar on the expression of downstream genes. These in-frame deletions in *mla* genes were introduced in SR23660 carrying chromosomal *lapC190* and a plasmid with an inducible expression of the *marA* gene by bacteriophage P1-mediated transduction. Transductants were plated on LA agar at 30 °C and at 43 °C in the presence of 75 μM IPTG to induce the expression of the *marA* gene (Table 4). As a control, *mlaC*, *mlaD* and (*mlaC mlaD*) in-frame deletions were introduced in the wild-type and *lapC190* bacteria. While such deletions could be introduced into the wild type, *lapC190* and SR23660 *lapC190* + p*marA*^+^ at 30 °C at a similar frequency; however, at 43 °C, deletion sets of *mla* genes could not be introduced in SR23660 even with the induction of the *marA* gene expression (Table 4). In control transductions, all three deletions of *mla* genes could be introduced in the wild type at 43 °C (Table 4). These results allow us to unambiguously conclude that *marA*-mediated multicopy suppression of *lapC190* bacteria requires the expression of *mla* genes. 

### 2.7. Absence of mla Genes Does Not Alter LPS Composition

**Figure 7:**
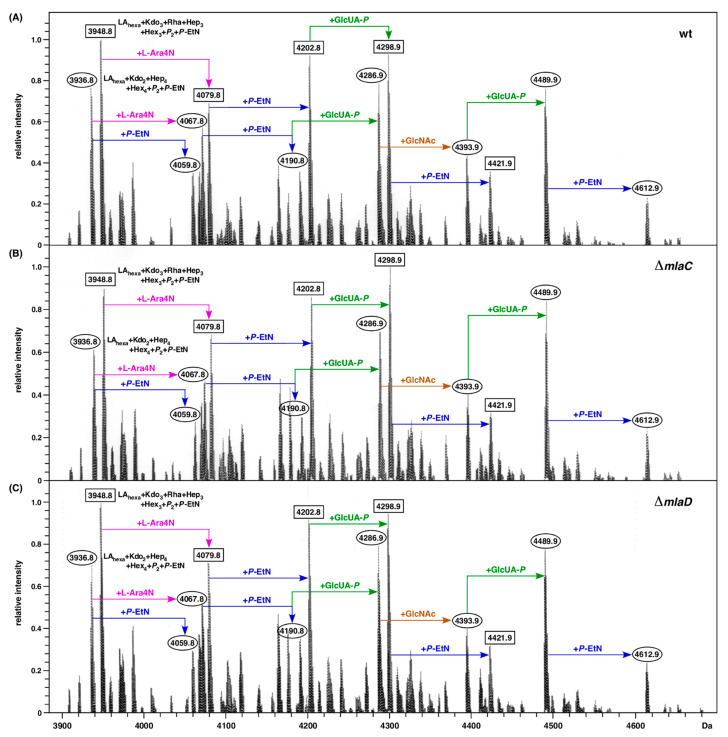
In the absence of either the *mlaC* or *mlaD* gene, LPS structure is similar to that of parental wild type. Charge-deconvoluted mass spectra in the negative ion mode of LPS from the wild type (**A**) and its Δ*mlaC* (**B**) and Δ*mlaD* (**C**) derivatives. Cultures were grown in phosphate-limiting medium at 30 °C. Mass numbers refer to monoisotopic peaks. Mass peaks with rectangular boxes correspond to the glycoform containing the third Kdo. Mass peaks marked with an oval box correspond to predicted LPS containing two Kdo residues.

In *E. coli*, up to now, no structural/compositional analysis of LPS has been reported when the Mla system is impaired. To investigate if the deletion derivatives of *mla* genes cause any major alterations in the LPS composition or in the incorporation of non-stoichiometric modifications, purified LPS obtained from the wild type and its isogenic derivatives were examined using mass spectrometric analysis. For such experiments, LPS was extracted after growth in phosphate-limiting growth medium as described earlier, since such growth conditions are inducive for lipid A and LPS core modifications due to the induction of BasS/R and PhoB/R two-component systems [13,43]. Examination of mass spectra of LPS obtained from the wild type and isogenic Δ*mlaC* and Δ*mlaD* derivatives shows similar mass peaks corresponding to the presence of complete glycoform I represented by mass peaks at 3936.7 Da and its derivatives with predicted incorporation of additional 1 or 2 P-EtN residues and also modification by L-Ara4N and GlcUA moieties as indicated. These mass peaks can be explained as LA_hexa_ (Kdo_2_Hep_4_Hex_4_P_2_) accompanied by additional substitutions with P-EtN and L-Ara4N, as shown (Figure 7). The mass peaks at 4489.9 Da and 4612.9 Da can be attributed to the addition of GlcNAc and GlcUA to glycoform I (Figure 7). These spectra from LPS obtained from either the wild type or its Δ*mlaC* and Δ*mlaD* derivatives also reveal a prominent presence of mass peaks corresponding to glycoforms IV and V, which are represented by mass peaks at 3948.7, 4079.8, 4202 and 4298.9 Da. These mass peaks are explained by the incorporation of a third Kdo and Rha linked to the Kdo disaccharide with a concomitant truncation of the outer core with a predicted composition of LA_hexa_ (Kdo_3_RhaHep_3_Hex_3_P_2_). The mass differences in these derivatives of glycoforms with a third Kdo and Rha are explained by the additional incorporation of P-EtN, L-Ara4N and GlcUA, as shown (Figure 7). The main findings from mass spectrometry data are that a deletion of *mla* genes does not alter LPS core and lipid A structural properties under these defined growth conditions.

### 2.8. SrrA-Mediated Suppression of lapC190 Mutant Bacteria Requires the Presence of Cardiolipin Synthase A and ClsA Is Essential for lapC190

Among various multicopy suppressors that were identified, SrrA has a distinguishing property that the corresponding gene in high dosage can suppress the Ts phenotype of strains lacking six cytoplasmic peptidyl-prolyl *cis*/*trans* isomerases, Δ*lapD* bacteria and is again reisolated as a suppressor of the Ts phenotype of *lapC190* bacteria [28,36]. Since a modest overproduction of SrrA was found to restore LpxC amounts (Figure 5), we further investigated a possible pathway that can explain this suppression. The same strategy of isolating transposon insertion mutants was used as shown above with the *marA* gene. Thus, the strain SR23951 carrying the chromosomal *lapC190* mutation and expressing the *srrA* gene from a tightly regulated P_T5_-*lac* promoter was used to serve as a host to generate a saturated library of Tn*10* Kan insertion mutants under permissive growth conditions at LA 30 °C. This library was next screened on LA agar supplemented with 75 μM IPTG to induce the expression of the *srrA* gene at 43.5 °C. Transposon mutants that exhibit a Ts phenotype even with the induction of the *srrA* gene were retained. Following bacteriophage P1-mediated transductions of such Tn*10* Kan mutants in the wild type, those conferring per se a Ts phenotype in the wild type were not followed. The identity of the Tn*10* insertion was obtained after DNA sequencing of inverse PCR products obtained from the template chromosomal DNA of the remaining Tn*10* insertion-carrying mutations, which specifically abrogated the multicopy suppression of the *srrA* gene in *lapC190* mutant bacteria. Analysis of DNA sequencing revealed that the majority of strains (8 out of 11) had Tn*10* insertion in the coding region of the *clsA* gene encoding cardiolipin synthase A. Two other strains had a mutation in the *cpxR* gene. CpxR acts as the DNA-binding response regulator and works with CpxA IM kinase, constituting one of the main two-component systems that responds to protein folding defects in the periplasm and severe defects in LPS biosynthesis [13,44,45,46,47]. One Tn*10* insertion mutant that did not allow the multicopy suppression by the *srrA* gene has an identical Tn*10* insertion in the heat shock promoter of the *groSL* operon at nt position -101, which was earlier found to abolish DksA-mediated suppression of Δ6*ppi* and Δ*dnaK*/*J* strains [36]. GroSL chaperonins constitute the major player in protein folding and are indispensable for bacterial viability [48,49]. This Tn*10* insertion in the *groSL* operon is located 3 nt downstream of the -35 heat shock promoter element, which is 101 nt upstream of the translational initiation codon as described previously with a reduced amount of GroL with its impaired induction at a high temperature [36]. Since the majority of Tn*10* insertion mutations mapped to the *clsA* gene, this suggests that the presence of cardiolipin is essential for SrrA-mediated restoration of the growth of *lapC190* mutant bacteria at elevated temperatures.

To further understand the requirement of the *clsA* gene when LapC function is impaired, a Δ*clsA* mutation was introduced in the wild type, *lapC190* with vector alone and *lapC190* + p*srrA*^+^ by bacteriophage P1-mediated transductions. Such transductants were subsequently verified for their growth at 43 °C. Results from such experiments show that Δ*clsA* is viable in the wild type under such growth conditions but cannot be introduced into *lapC190* bacteria with a vector alone (Table 5). However, transductants at a highly reduced frequency with a smaller colony size were obtained in SR23951 *lapC190* + p*srrA*^+^ in the presence of IPTG at only 30 °C as compared to a wild-type background. Furthermore, Δ*clsA* derivatives in SR23951 were unable to propagate at temperatures above 42 °C (Table 5). Thus, these results show that ClsA presence is required for SrrA to act as a multicopy suppressor to allow the growth of *lapC190* bacteria at high temperatures and presumably ClsA and LapC cooperate in the maintenance of phospholipid homeostasis in the cell envelope.

### 2.9. SrrA Does Not Regulate Transcription of the clsA Gene

**Figure 8:**
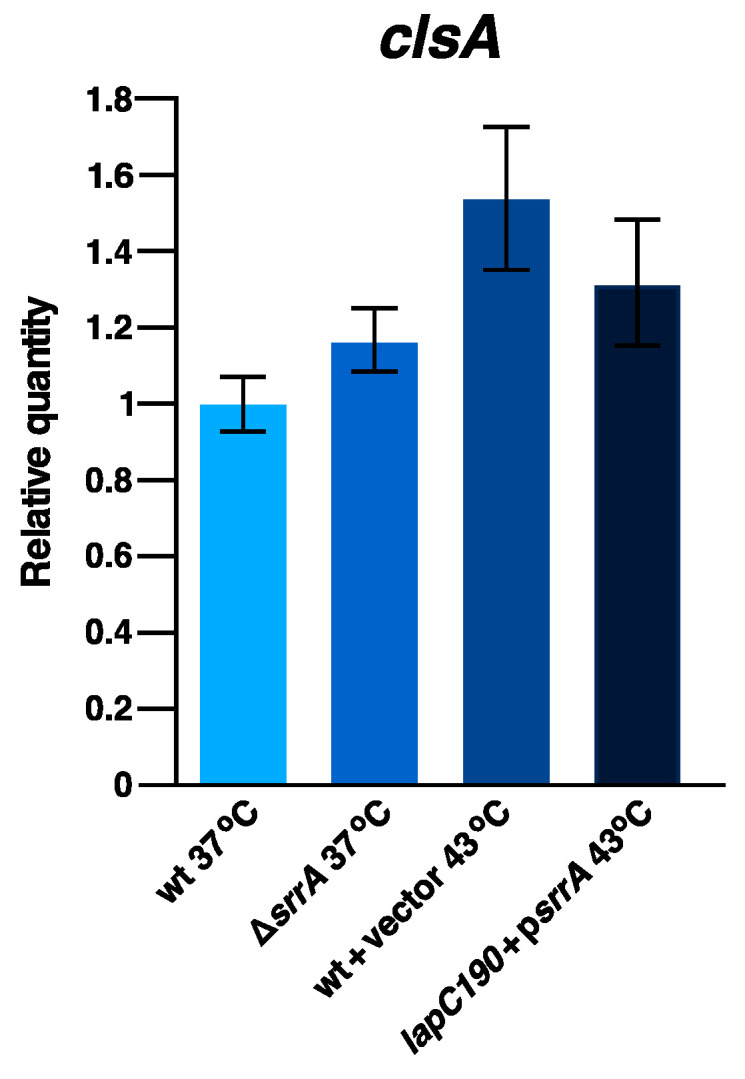
Transcription of the *clsA* gene is not regulated by SrrA. qRT-PCR analysis of mRNA isolated from wild-type bacteria, its isogenic derivative Δ*srrA*, grown up to an OD_595_ of 0.2 in LB medium at 37 °C. For heat shock experiments isogenic cultures of wild type with empty vector and *lapC190* bacteria carrying the inducible *srrA* gene on a plasmid were grown at 30 °C in M9 minimal medium up to an OD_595_ of 0.2 and supplemented with 75 μM IPTG prior to a 15-min shift to 43 °C. Data presented are from RNA isolated from three biological replicates and error bars are indicated.

SrrA is predicted to act as a transcriptional regulator since it contains an N-terminal signal recognition Per-Arnt-Sim (PAS) domain and a C-terminal helix-turn-helix motif [36]. However, the genes whose expression is regulated by SrrA are unknown. As described above, the overexpression of the *srrA* gene efficiently suppresses the Ts phenotype of *lapC190* bacteria and also either of the strains collectively lacking six cytoplasmic peptidyl-prolyl *cis*/*trans* isomerases or of Δ*lapD* bacteria [28,36]. Since SrrA-mediated suppression requires the presence of a functional *clsA* gene, we undertook studies to address if SrrA acts by regulating transcription of the encoding gene. Thus, total RNA was extracted from the wild type and its isogenic derivative Δ*srrA* from cultures grown at 37 °C. In parallel, total RNA was also extracted from wild-type bacteria carrying either an empty vector or expressing the *srrA* gene from an inducible promoter. For such experiments, cultures were grown at 30 °C. Prior to heat shock, 75 μM IPTG was added to induce transcription of the *srrA* gene, and cultures were shifted to 43 °C for 15 min. The equivalent amount of total RNA from these four sets of RNAs was next subjected to q-RT-PCR analysis using *clsA*-specific oligonucleotides for the synthesis and quantification of cDNA. The quantification of the *clsA* transcription pattern showed nearly similar levels of *clsA* transcripts between the wild-type and Δ*srrA* bacteria at 37 °C (Figure 8). Similarly, when the *srrA* gene expression was induced and when RNA was extracted from 43 °C and the relative abundance of transcripts were estimated by q-RT-PCR, no significant differences were observed in the *clsA* expression with or without *srrA* induction (Figure 8). Although a minor increase in *clsA* mRNA can be observed upon shift to 43 °C as compared to the relative abundance at 37 °C, such an increase is not comparable to that of heat shock genes. Thus, results from q-RT-PCR allow us to conclude that the *clsA* transcription is not regulated by SrrA either at 43 °C or at 37 °C, and overexpression of the *srrA* gene or its absence does not alter the abundance of *clsA* transcripts.

### 2.10. LapD-Mediated Suppression of a lapC190 Mutant Bacteria Requires LapD N- and C-Terminal Domains and Identification of Some of the Critical Amino Acid Residues Required for LapD Function

**Figure 9:**
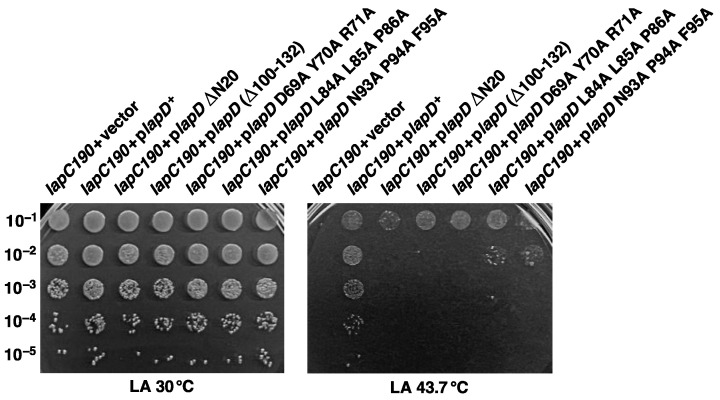
The N-terminal membrane anchor of LapD and its conserved amino acid residues in the C-terminal domain are essential for the restoration of the growth of *lap190* bacteria at 43.7 °C. In-frame deletion of N-terminal 20 amino acid residues, ΔN20, deletion of last 32 amino acid residues Δ100-132 and a triple Ala replacement of conserved amino acid were constructed by gene synthesis and cloned in expression vector. Plasmid DNA with the wild-type *lapD* gene and plasmids DNAs with various substitutions or deletions in the *lapD* gene were used to transform *lapC190* bacteria. Growth of such isogenic cultures of *lap190* was quantified by serial spot dilution assay on LA 30 °C and 43.7 °C. The relevant genotype and temperature of incubation are indicated.

As overexpression of the *lapD* gene restored the growth of *lapC190* mutant bacteria at a high temperature, we sought to address if specific domains of LapD are required for its function. LapD has a single N-terminal transmembrane anchor and a highly conserved C-terminal domain. The crystal structure of the LapD counterpart in *Haemophilus ducreyi* shows a unique tetrameric α-helical coiled-coil structure in the cytosolic domain, which is predicted to mediate protein–protein interactions [50]. Thus, a series of point mutations (substitution of conserved residues by Ala), deletion of the N-terminal membrane anchor (1–20 amino acids) and deletion of the C-terminal last 32 amino acids were constructed. Among the Ala substitutions, we replaced conserved amino acids D69, Y70 and R71 with three Ala residues. Similarly, L84, L85 and P86 and N93, P94 and F95 were also substituted with three Ala residues in each case. All generated constructs with in-frame mutations were expressed under a tightly regulated *ptac* promoter and, when required, in-frame with an N-terminal hexa-His tag, where the expression is driven from a controlled T7 promoter. Plasmids where the expression is driven from the *ptac* promoter were transformed either into the Δ*lapD* strain or in *lapC190* bacteria and evaluated for the ability to suppress their Ts phenotype. Results from such experiments show that while the control plasmid overexpressing the wild-type *lapD* gene restores growth of *lapC190* at elevated temperature, neither the N-terminal nor the C-terminal deletions were able to suppress the Ts phenotype when the expression was induced by the addition of 75 μM IPTG (Figure 9). Similarly, three plasmid sets expressing replacement by Ala substitutions of amino acids (69–71), (84–86) and (93–95) were also unable to restore the growth of *lapC190* bacteria at elevated temperatures (Figure 9). These results allow us to conclude that specific conserved residues in the cytoplasmic domain are essential for LapD function. Furthermore, the single N-terminal membrane anchor and last 32 amino acid residues of the cytoplasmic domain are required for the *lapD* gene to act as a multicopy suppressor.

### 2.11. Multicopy Suppression of lapC190 Bacterial Defects Requires DksA’s PPIase and Transcriptional Activities

**Figure 10:**
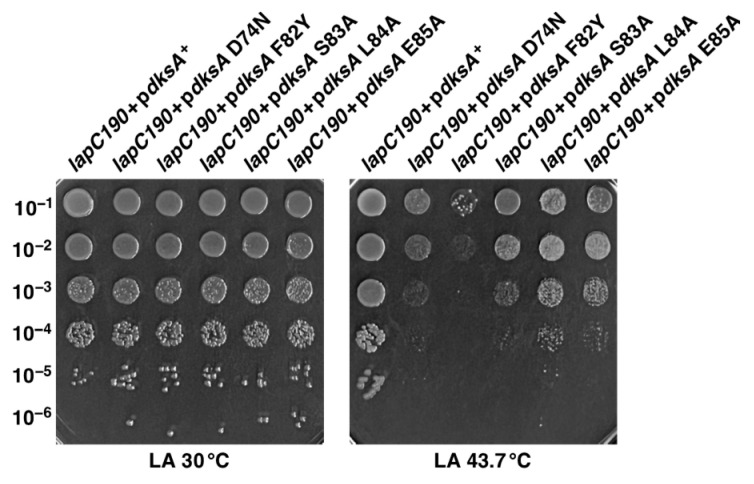
Amino acid residues that are essential for DksA’s transcriptional activity and those required for its peptidyl-prolyl *cis/trans* isomerase activity are required for its multicopy suppressing ability of *lapC190* bacteria. Competent cells of *lapC190* bacteria were transformed by plasmid DNA carrying either the wild-type *dksA* gene or with specific single-amino amino acid replacement in the tip domain of the coiled-coil domain of DksA. Cultures of such isogenic cultures of *lap190* bacteria were grown at 30 °C in LB medium and bacterial growth was quantified by serial spot dilution assay on LA 30 °C and 43.7 °C. The relevant genotype and temperature of incubation are indicated.

The results presented in the above sections have shown that overexpression of the global transcriptional regulator encoded by the *dksA* gene can restore the bacterial growth of *lapC190* bacteria at elevated temperatures and also suppress LpxC degradation defects. We have earlier shown that DksA also suppresses the Ts phenotype of Δ6*ppi* bacteria and Δ*lapD* bacteria [28,36]. Characterization of the biochemical properties of DksA further revealed that besides acting as a global transcriptional regulator, it also exhibits the PPIase activity, which can be inhibited by the FK506 macrolide [36]. Several studies have led to the conclusion that the coiled-coil domain of DksA inserts into the secondary channel of RNAP and that residues at the tip of the coiled-coil domain of DksA are important for its activity [51,52,53]. More recent studies suggest that amino acid residues in this coiled-coil domain play an essential role for the transcriptional and PPIase activities of DksA [36]. Thus, to examine which activity of DksA is required for the suppression of growth defects of *lapC190* bacteria, cloned DNA of D74N, F82Y, S83A, L84A and E85A variants of the *dksA* gene were transformed into *lapC190* bacteria, using wild-type p*dksA*^+^ as a control. It is important to mention that D74, F84, S83 and E85 amino acid residues are important for DksA-mediated transcriptional regulation of reporter genes such as the *rrnB*P1 promoter [36]. However, the replacement of the F82 amino acid residue by the Tyr residue abolishes the PPIase activity of DksA, although such a variant behaves like wild type concerning the regulation of the *rrnB*P1 promoter [36]. Our complementation experiments reveal that cloned D74N, F82Y, S83A, L84A and E85A mutants of the *dksA* gene have all lost the ability to fully restore the growth of *lapC190* bacteria at high temperatures upon their overexpression as compared to the complete suppression of Ts phenotype by the wild-type *dksA* gene (Figure 10). Viable colonies at all dilutions, albeit with smaller colony size, are observed when D74N, S83A, L84A and E85A mutants of the *dksA* gene were overexpressed in a *lapC190* strain (Figure 10). However, replacement of F82 by Y, which is a critically important residue for DksA’s PPIase activity, completely abrogates the DksA-mediated multicopy suppression of *lapC190* bacteria (Figure 10). These results suggest that the PPIase activity of DksA is most critical for DksA to function as a multicopy suppressor to suppress the Ts phenotype of *lapC* mutant bacteria, and the transcriptional role of DksA, although important, could only play a minor role. This is particularly striking due to the abrogation of suppression by overexpression of the DksA F82Y variant in comparison to only a partial loss of function with other DksA mutants concerning the LPS-related function.

## 3. Discussion

In this study, we identified new additional factors that regulate LpxC levels. Regulation of LpxC is critical for our understanding of the mechanism of balancing biosynthesis of LPS and phospholipids, since they both share the same (*R*)-3-hydroxymyristate-ACP as the common metabolic precursor. Recent studies have shown that LpxC stability is regulated by the FtsH-LapB complex, wherein FtsH degrades LpxC, which requires LapB function for its proteolytic activity towards LpxC [19,22]. However, this activity is counteracted by the essential LapC (YejM) protein [6,23,25]. In addition to FtsH-LapB and LapC, many additional factors participate in the regulation of LpxC amounts and, hence, the overall LPS content. Mutations in either the *ftsH* or *lapB* genes lead to an increased abundance of LPS due to the stabilization of LpxC, whereas loss-of-function mutations in the *lapC* gene exhibit enhanced LpxC degradation with a concomitant reduction in the overall LPS content. During earlier work, we isolated temperature-sensitive *lapC190*, *lapC377fs* and *lapC* F349S mutant bacteria [6]. Because *lapC190* mutant bacteria exhibit a tight Ts phenotype, we exploited such a property to isolate multicopy suppressors that restore growth at elevated temperatures to identify additional factors that regulate LpxC amounts. Such an approach should also reveal what are the limiting factors that confer the Ts phenotype to *lapC190* mutant bacteria. Using such a procedure, thirteen genes were identified whose mild induction overcomes the Ts phenotype. As *lapC190* bacteria exhibit permeability defects and reduced LpxC and LPS levels, we examined whether overexpression of any of these genes also suppress such defects. Quite satisfactorily, a major proportion of such genes can be implicated either directly or indirectly in functions related to LPS, phospholipoid/fatty acid biosynthesis and regulation of LpxC protease FtsH amount (*lapD*, *pldA*, *acpP*, *acpT*, *accB*, *gnsA*, *yfgM*, *marA*) or envelope stress response (*srrA*, *dksA, ymgG*). These thirteen genes were thus grouped into different categories based on their known or predicted functions. Three out of them (*marA*, *dksA* and *srrA*) act as transcriptional factors and should contribute by their regulation of downstream target gene(s), whose gene products either directly regulate LPS or phospholipid biosynthesis. Another group of suppressors include genes encoding the LPS assembly protein LapD, the essential AcpP protein that shuttles fatty acid chains in the lipid A and fatty acid/phospholipid biosynthesis, the outer membrane phospholipase A encoded by the *pldA* gene. Isolation of these genes is interesting since this shows that *lapC* mutant bacteria have imbalanced phospholipid and LPS biosynthesis. Importantly, PldA degrades phospholipids that have been mislocalized to the outer leaflet in the OM, which signal stabilization of LpxC and an increased production of LPS [29], explaining its isolation as a multicopy suppressor. However, it should be noted that overexpression of the *pldA* gene does not overcome the permeability defects of *lapC190* bacteria, as reflected by an inability to restore growth on bile-salt-containing MacConkey agar. Interestingly, up to now, the sole dosage-dependent suppressor of *lapC* mutant bacteria expressing only the periplasmic domain has been the *acpT* gene, whose product encodes a phosphopantetheinyl transferase [54]. In this work, we also isolated the *acpT* gene as a multicopy suppressor; however, this suppressing phenotype was much weaker than the others. Surprisingly, it has been shown that AcpT-mediated suppression of *lapC* (*yejM*) mutant bacteria for the Ts phenotype does not require its enzymatic activity [54].

The global transcriptomes of MarA and DksA are known [55,56,57], but only sketchy information about SrrA is available. Thus, in this study, we systematically attempted to identify genes whose presence is required for multicopy suppression by MarA and SrrA. In *E*. *coli*, MarA acts as a transcriptional activator and is known to activate the expression of *acrAB*-*tolC*-encoded efflux pump, thereby contributing to resistance against antimicrobial compounds [58,59,60]. More recently, approximately 30 transcriptional units have been identified that could be regulated by MarA [61]. Here, we used saturated transposon mutagenesis to identify genes whose inactivation abrogates the *marA-* and *srrA*-mediated restoration of the growth of *lapC190* mutant bacteria at high temperatures. The vast majority of transposon mutants that prevented MarA-mediated multicopy suppression mapped to the *mla* operon, whose products are implicated in lipid trafficking, leading to the removal of the mislocalized phospholipids from the OM [29]. Consistent with our results, a MarA-binding motif has been identified in the promoter region of the *mla* operon [61]. Thus, our results provide a rationale for the identification of the *marA* gene as a multicopy suppressor of *lapC190* mutant bacteria, and this suppression requires the functionality of *mla* genes. In accordance with these findings, the *pldA* gene was isolated as a multicopy suppressor since both PldA and Mla pathways sense OM disturbance due to the accumulation of phospholipids in the OM when LPS is limiting.

In a similar manner, we addressed the pathway of SrrA-mediated suppression. Our results show that the overexpression of the *srrA* gene efficiently restores the growth at high temperatures, LpxC levels as well as permeability defects. This suppression was found to be contingent on the presence of the functional copy of the *clsA* gene encoding cardiolipin synthase A and the envelope-responsive two-component system CpxA/R. The CpxA/R system, along with the RpoE sigma factor, responds to severe defects in LPS assembly and biosynthesis as well as protein misfolding in the periplasm [13,19,39,62]. We have previously shown that ClsA is required for the viability of strains synthesizing either penta- or tetraacylated lipid A, or when LapD assembly protein is missing [15,28]. Since LPS is presumably sensed in the inner membrane by LapC, the absence of ClsA could further accentuate this defect as it is required to assist MsbA-mediated LPS translocation. However, this requires further studies because the *clsA* gene is per se essential in *lapC190* bacteria. Our gene expression studies did not show that SrrA regulates transcription of the *clsA* gene based on q-RT-PCR results. Thus, we are initiating a global transcriptosome study to identify genes that are regulated by SrrA, and initial results, which require further in-depth experimentation, suggest that SrrA acts as a transcriptional repressor of the envelope stress response and fatty acid metabolism in *E*. *coli*.

Another important finding from this study is the partial overlap between LapD and LapC functions in the regulation of LpxC and LPS levels. This is based on the following results: (i) Some of the multicopy suppressors identified in this work that relieve the Ts phenotype of *lapC190* bacteria were earlier also identified as suppressors of Δ*lapD*. These include *dksA*, *srrA*, *acpP*, *accB* and *yfgM* [28]. (ii) Of significance is the isolation of the *lapD* gene as a multicopy suppressor of *lapC* mutant bacteria for the Ts phenotype. We recently proposed that LapD could also act upstream of LapB-FtsH in a manner similar to LapC, since suppressors mapping to *lapB*, *ftsH* and *lpxC* that stabilize LpxC, can suppress Δ*lapD* and *lapC190* bacteria. (iii) However, some suppressors, like the *marA* gene and the *acpT* gene cannot relieve the Ts phenotype of Δ*lapD* bacteria upon their overexpression and are specific to *lapC* mutant bacteria. Consistent with these results, the overexpression of the *lapC* gene does not relieve the Ts phenotype of Δ*lapD* bacteria. These results are not surprising because, at the biochemical level, despite the LapC and LapD co-purification with LapB, several other interacting partners of LapD are unique to it and were not found to be part of the LapC complex [6,24,28].

To further understand LapD-mediated multicopy suppression of *lapC* mutant bacteria, we constructed a series of mutations in the *lapD* gene to identify the essential domains of the encoded protein. This allowed us to express such mutant versions in a controlled manner from a regulated inducible promoter in *lapC* mutant bacteria. This revealed that the N-terminal membrane anchor and the last 32 conserved C-terminal amino residues are critical for LapD function. Furthermore, several residues that were predicted for LapD to interact with its partners [50] were also found to be essential for its function since their replacement by Ala amino acid residues abolished LapD function (inability to suppress the Ts phenotype of either Δ*lapD* or *lapC190* mutant bacteria). This includes a motif constituted by the N93 P94 F96 triplet, which we show is essential for the LapD function and has been predicted to interact with LapA. This conserved NFP motif is located after the long cytoplasmic helical domain. 

Regarding the mechanism of suppression by overproduction of the global transcriptional regulator DksA, we show that, most critically, its PPIase activity is required for this activity. However, the replacement of conserved amino acid residues in the tip region of the coiled-coil domain of DksA which are known to be essential for its role in interaction with RNA polymerase and hence the transcriptional regulation caused a highly attenuated ability to confer suppressing ability of *lapC190* bacteria. However, the most severe phenotype was the replacement of F82 by Y, which resulted in the total abolishment of the multicopy-suppressing ability of DksA. This amino acid residue is important for conferring the PPIase activity of DksA but is not essential for transcriptional function [36]. Hence, the PPIase activity of DksA is critical for its multicopy-suppressing ability, and its transcriptional activity also plays an important role. Further studies are required to determine the mechanism(s) of the restoration of growth at elevated temperatures by other multicopy suppressors that were identified in this study.

It would be interesting to determine whether the suppression by *gnsA* overexpression is due to an increase in the unsaturated fatty acid synthesis that could lead to the stabilization of LpxC. The *gnsA* gene was initially identified as a multicopy suppressor of the *secG* null mutant and was shown to increase the acidic phospholipid content and inhibit phosphatidylethanolamine accumulation via an unknown mechanism [63]. Furthermore, overexpression of the *gnsA* gene also suppresses the Ts phenotype of *fabA6* mutation and causes an increase in the unsaturated fatty acid content, such as *cis*-vaccenic acid (18:1), at the expense of palmitic acid (16:0), particularly at low temperatures [64]. At present, the molecular basis of the suppression of either *lapC190* bacteria or the *fabA6* Ts mutation or the ascribed increase in the unsaturated fatty acid content is not known. To this end, we have started to measure the impact of *gnsA* overexpression on fatty acid and phospholipid content in various *lapC* mutant bacteria. It is also intriguing why the central cofactor acyl carrier protein (AcpP) is limiting in *lapC190* bacteria. AcpP is the fourth most abundant protein in *E*. *coli*, its excess is usually toxic in wild-type bacteria [65], and yet its mild induction quite effectively overcomes the various growth defects of *lapC* mutant bacteria without increasing LpxC stability or restoring LPS amounts. AcpP is involved in not only LPS and phospholipid biosynthesis, but its interactome contains more than 35 proteins, some of which are unrelated to LPS and fatty acid synthesis [66]. Since the replacement of catalytically active site residues confers a toxic growth phenotype due to the accumulation of *apo*-ACP [67], we could not address if the catalytic activity of AcpP is required for the suppressing phenotype. However, AcpP overproduction does not enhance either LPS or LpxC amounts, and thus, its action could be either rebalancing fatty acid composition or via some another mechanism. Isolation of other multicopy suppressors, such as the *yfgM* gene, can be rationalized, since its product has been found to be a substrate of FtsH protease [68]. Overexpression of the *yfgM* gene could potentially titrate out FtsH, and this could lead to the stabilization of LpxC. The function of YceJ is currently unknown. However, the expression of the *yceJ* gene is induced by a variety of stress conditions such as heat shock, the induction of extracytoplasmic responsive CpxR regulon and when the Lol system (lipoprotein sorting) is inhibited [69]. Thus, YceJ may be involved in stress response management that could directly or indirectly modulate LpxC levels and hence more studies are required to address its function.

In summary, in this study, we identified new proteins, such as the transcriptional activator MarA, factors that are required to maintain OM asymmetry (PldA, Mla system), and the essentiality of cardiolipins which participate in the regulation of LPS assembly summarized in the Figure 2. To date, MarA has not been shown to directly regulate LpxC stability, which we have now established here. Isolation of the *srrA* gene as a multicopy suppressor is interesting because its overexpression stabilizes LpxC. We also identified essential amino acid residues that are required for LapD function and its partial overlap with LapC in regulating LpxC amounts.

## 4. Materials and Methods

### 4.1. Bacterial Strains, Plasmids and Media

The various bacterial strains and plasmids used in this study are described in Table 6. Luria-Bertani agar (LA), LB broth (Difco, Franklin Lakes, NJ, USA), M9 minimal media and MacConkey agar (Difco) were prepared as described previously [13,19]. Growth medium was supplemented with antibiotics kanamycin (30 or 50 μg/mL), chloramphenicol (10 or 30 μg/mL) and ampicillin (100 μg/mL). The strain carrying the *lapC190* mutation in BW25113 has been previously described and when required was transduced into different genetic backgrounds using bacteriophage P1-mediated transductions at 30 °C [6]. Non-polar Δ*marA* and Δ*mlaCD* mutations were constructed by using the λ Red recombinase mediated recombination system [70]. The kanamycin resistance cassette was amplified using pKD13 as a template [70]. PCR products from such amplification reactions were electroporated into BW25113 derivative GK1942 containing the λ Red recombinase-encoding plasmid pKD46. The *aph* cassette was removed using the plasmid pCP20 expressing FLP recombinase at 30 °C and verified to have lost the antibiotic marker by streaking on LA plates with an appropriate antibiotic. The construction of some of the deletion derivatives used in this study, Δ*dksA*, Δ*srrA*, Δ*clsA*, Δ*lpxL*, Δ*lpxM* and Δ*lapD* has been previously described [13,28,36]. Isogenic deletion combinations were constructed using bacteriophage P1-mediated transductions at 30 °C on either the LA or M9 medium.

### 4.2. Identification of Multicopy Suppressors Whose Overexpression Suppresses the Temperature-Sensitive Phenotype of lapC190 Bacteria

As *E*. *coli* K-12 strains carrying the chromosomal *lapC190* mutation exhibit the Ts phenotype (inability to grow above 42 °C), we used such a growth defect to identify genes whose overexpression can allow growth under such non-permissive conditions. To achieve this, competent cells of SR23529 and SR23583 *lapC190* derivatives of BW25113 were transformed with a complete genomic library of all predicted ORFs of *E*. *coli* cloned in pCA24N (ASKA collection). Transformants were selected at 43 °C in the presence of IPTG (75 μM). In this library, the expression of an individual gene is driven from the tightly regulated IPTG-inducible P_T5_-*lac* promoter. The concentration of IPTG used in this study has been optimized to provide a mild induction of genes without any toxicity [19]. Cultures of such Ts^+^ colonies were grown to obtain plasmid DNAs and used to retransform parental *lapC190* mutant bacteria. Transformants were plated on growth medium in the presence of IPTG at 43 °C and 43.5 °C, and plasmids which allowed restoration of growth were retained. DNA insert of all relevant plasmids that restored the growth upon retransformation on LA-rich medium at 43.5 °C was sequenced.

### 4.3. The Isolation of Chromosomal Transposon Insertion Mutations That Prevent the Suppression by Overexpression of Either the marA Gene or the srrA Gene

As overexpression of either the *marA* gene or the *srrA* gene restore the growth of *lapC190* bacteria, chromosomal Tn*10* transposon insertions were isolated to understand the mechanism of their suppression as previously described [36]. Thus, the strains SR23660 and SR23951 *lapC190* derivatives, which can grow at 43.5 °C carrying either the *marA* gene or the *srrA* gene, respectively, were used as a host to isolate Tn*10* insertions. In these strains, suppressing *marA* and *srrA* genes are cloned in pCA24N vector with expression regulated by P_T5_-*lac* promoter. For the mutagenesis, SR23660 (*lapC190* + p*marA*^+^) and SR23951 (*lapC190* + p*srrA*^+^) isogenic strains served as recipients using λTn*10* kan bacteriophage to obtain random saturated transposon insertions. More than 50,000 transposon insertion mutants were isolated in each strain background in LA medium at 30 °C (permissive growth conditions) as described previously [36]. Transductants were screened for a Ts phenotype on LA medium at 43.5 °C supplemented by 75 μM IPTG to induce the expression of *marA* and *srrA* genes. Transductants that were unable to grow at 43.5 °C were retained and a bacteriophage P1 was grown on them individually. Such bacteriophage P1 lysates were used to retransduce SR23660 and SR23951 strains. Transductants were tested for their inability to grow at 43.5 °C and those which bred true (Ts phenotype and hence lack suppression) were further used. Such transposon mutants were next transduced into the wild-type strain BW25113 to identify a transposon insertion conferring the Ts phenotype in the wild-type background. Generally, only those Tn insertions that did not allow multicopy suppression by either the *marA* gene or the *srrA* gene were further used to obtain chromosomal DNA to identify the position of Tn*10* insertion. The position of Tn*10* was determined by the inverse PCR with nested primers and sequenced using the Tn*10* primer as described earlier [19].

### 4.4. Estimation of LpxC Amounts by Immunoblotting

The isogenic bacterial cultures of wild type, its derivative with the *lapC190* mutation with the vector alone and its isogenic cognates carrying multicopy suppressor encoding genes were grown in LB medium at 30 °C. Exponentially grown cultures were adjusted to an OD_595_ of 0.05 and allowed to further grow up to an OD_595_ of 0.2. For the induction of expression of the suppressing gene, IPTG at the final concentration of 75 μM was added and shifted in prewarmed flasks held at 43 °C for 2 h. Cultures were harvested by centrifugation at 10,000 rpm for 15 min and pellets were solubilized in the sample buffer. The protein concentration of each sample was measured using the Pierce BCA protein assay kit (Thermo Scientific, Waltham, MA, USA). Equivalent amounts of proteins were applied to a 12% SDS-PAGE and transferred by Western blotting. Blots were probed with polyclonal antibodies against LpxC, or with anti-TrxA as a loading control as described earlier [6]. Blots were revealed by a chemiluminescence kit from Thermo Scientific as per the manufacturer’s instructions.

### 4.5. Site-Directed Mutagenesis of the lapD Gene and In Vivo Complementation Analysis

To identify amino acid residues and specific domains in LapD, PCR and Gibson cloning was used. Firstly, two sets of deletions of the *lapD* gene were constructed using oligonucleotides for PCR amplification to clone only the region encoding either the soluble domain or removal of the coding region for the last 32 C-terminal amino acid residues. PCR products were cloned in the pCA24N vector where the expression is tightly regulated from P_T5_-*lac* promoter and for the protein induction in T7 promoter-based pIVEX 4.2 vector (Roche diagnostics, Basel, Switzerland). Three specific sets of mutations (replacement of D69, Y70 and R71 with Ala substitutions in the coding region of the *lapD* gene were introduced by using gene synthesis and Gibson cloning. Similarly, L84, L85 and P86 were substituted by three Ala, and in the third set, N93, P94 and F95 were also replaced with Ala residues. In the three resulting plasmid sets, the expression of mutated *lapD* gene is regulated from P_T5_-*lac* promoter. For in vivo complementation, Δ*lapD* and *lapC190* mutant bacteria were transformed by empty vector and plasmid expressing the wild-type gene and where the *lapD* gene is mutated. Transformants were plated on LA agar at 30 °C. Cultures of such transformed derivatives subsequently were grown to an exponential growth phase and growth properties measured by spot dilution assays on LA medium at 30 °C and 43.5 °C.

### 4.6. LPS Extraction, Mass Spectrometry and Measurement of LPS Levels

Typically, 400 mL cultures of wild type, its isogenic Δ*mlaC*, Δ*mlaD* and Δ(*mlaC mlaD*) derivatives were grown in phosphate-limiting medium at 37 °C. Cultures were harvested by centrifugation at 10,000 rpm for 30 min and the pellets were lyophilized. LPS was extracted by the phenol/chloroform/petroleum ether procedure [72]. For the LPS analysis, lyophilized material was dispersed in water by sonication and resuspended at a concentration of 2 mg/mL. For mass spectrometric data acquisition, LPS samples were dissolved at a concentration of ~10 ng/μL and analyzed as described previously [13,73]. Electrospray ionization-Fourier transform ion cyclotron (ESI-FT-ICR)-mass spectrometry was performed on intact LPS in the negative ion mode using an APEX QE (Bruker Daltonics, Breman, Germany) equipped with a 7-tesla actively shielded magnet and dual ESI-MALDI. Mass spectra were charge deconvoluted, and mass numbers given refer to the monoisotopic peaks.

For the estimation of LPS levels, an equivalent amount of bacterial cultures (wild type, its isogenic *lapC190* derivative and its derivatives carrying various multicopy suppressor-encoding genes) were grown up to an OD_595_ of 0.5. Cultures were harvested by centrifugation and pellets were resuspended in 1X sample buffer. To obtain whole cell lysates, samples were boiled for 10 min, followed by digestion with Proteinase K. Equivalent portions of obtained whole cell lysates were applied to a 15.5% SDS-Tricine gel. After the electrophoresis, LPS was transferred by Western blotting. Immunoblots were probed for LPS amounts using the WN1 222-5 monoclonal antibody as described previously [6]. The WN1 222-5 monoclonal antibody [74] was used at a dilution of 1:10,000. Blots were revealed using a chemiluminescence kit from Thermo Scientific as per the manufacturer’s instructions.

### 4.7. RNA Purification and qRT-PCR Analysis

Exponentially grown cultures of the wild-type strain BW25113, its Δ*srrA* derivative SR19957, SR19761 (BW25113+vector pCA24N) and SR14857 (BW25113+p*srrA*^+^ in pCA24N) were used to extract total RNA. For comparison of wild type and its Δ*srrA* derivative, cultures were grown at 37 °C in LB and adjusted to an optical density OD_600_ of 0.05. Cultures were allowed to grow up to an OD_595_ of 0.2 and were then harvested by centrifugation. To quantify changes in the mRNA abundance when the expression of the *srrA* gene is induced, the bacterial culture of SR19761 and SR14857 were grown at 30 °C in LB-rich medium to an OD_595_ of 0.1. Prior to heat shock, IPTG at the final concentration of 75 μM was added. Aliquots were immediately shifted to a prewarmed medium held at 43 °C and incubated for another 15 min. Equivalent amounts of culture samples were collected from cultures grown at 30 °C and after the heat shock of 43 °C, they were harvested by centrifugation. Total RNA was extracted using hot phenol extraction as described previously [75]. Purified total RNA was treated RQ1 DNase (Promega, Madison, WI, USA) to remove any genomic DNA and ethanol precipitated. Pellets were resuspended in DEPC-treated water. RNA was subjected to another round of purification using the GeneElute universal total RNA isolation kit from Sigma Aldrich, St. Louis, MO, USA. RNA amounts were quantified and their integrity verified using agarose gel electrophoresis. q-RT-PCR was used to quantify changes in the *clsA* gene expression, using gene-specific primers. Two μg of purified mRNA was converted to cDNA using Maxima H-Minus Reverse Transcriptase (Thermo Scientific). Reactions were conducted for 40 cycles using PowerUp SYBR^®^ Green PCR Master Mix (Thermo Scientific), as described previously [6]. Q-RT-PCR was performed using the CFX Connect Real-Time PCR Detection System (Bio-Rad, Warsaw, Poland). Data were analyzed by software Bio-Rad CFX Maestro 2.3.

### 4.8. Bacterial Growth Analysis

For the measurement of relative restoration of growth by overexpression of different genes identified in this work, growth ability was quantified. For such experiments, exponentially grown cultures grown at 30 °C were adjusted to an optical density OD_595_ of 0.1, and ten-fold dilutions were spot tested on LA agar plates or on MacConkey agar at different temperatures in the presence of 75 μM IPTG to induce the expression of the suppressing gene present on the plasmid. Five μL of each dilution were spotted and the bacterial growth was analyzed after incubation for 24 h at 30 °C and 43 °C or at 43.5 °C as indicated in specific experiments.

## Figures and Tables

**Table 1:** Identification of the multicopy suppressors of *lapC190* bacteria that restore the growth at high temperatures and on MacConkey agar.

Growth Conditions				
Gene	LA 43 °C	MacConkey Agar 37 °C	Δ*lapD*	Function
*yfgM*	+	+	+	FtsH substrate, ancillary SecYEG translocon subunit
*dksA*	+	+	+	RNA polymerase-binding transcription factor
*artJ*	+	+	+	L-arginine ABC transporter periplasmic binding protein
*acpP*	+	+	+	acyl carrier protein
*accB*	+	-	+	biotin carboxyl carrier protein
*lapD*	+	ND	+	LPS assembly protein
*pldA*	+	-	-	outer membrane phospholipase A
*marA*	+	±	-	DNA-binding transcriptional dual regulator
*yceJ*	+	-	-	putative cytochrome b561
*gnsA*	+	-	-	putative phosphatidylethanolamine synthesis regulator
*ymgG*	+	-	±	PF13436 family protein toxin-antitoxin system
*srrA*	+	+	+	transcription (stress response regulator A)
*acpT*	+	+	-	*holo*-(acyl carrier protein) synthase 2

ND denotes not determined. Sign + indicates restoration of growth, - indicates lack of growth and ± refers to partial restoration of growth.

**Table 2:** Synthetic lethal combinations reveal essentiality of *marA*, *srrA* and *lapD* genes in *lapC190* bacteria.

Numbers of Transductants on LA 30 °C		
Donor	Wild Type	*lapC190*
Δ*marA*	1274	92 ^1^
Δ*srrA*	650	2
Δ*dksA*	>2000	163 ^2^
Δ*pldA*	1460	34 tiny
Δ*lpxL*	742	6
Δ*lpxM*	960	12
Δ*lapD*	733	25

^1^ not viable. ^2^ viable but heterogenous.

**Table 3:** Synthetic lethal combinations reveal essentiality of *lpxL* and *lpxM* genes in *lapC190* bacteria, which can be overcome in the presence of ectopic plasmid containing a wild-type copy of either of the genes.

Numbers of Transductants on LA 30 °C		
Donor	Wild Type	*lapC190*
	wild type + p*lpxL*	*lapC190* + p*lpxL*
P1 Δ*lpxL*	1234	1164
	wild type + p*lpxM*	*lapC190* + p*lpxM*
P1 Δ*lpxM*	968	1005
	wild type + p*lapD*	*lapC190* + p*lapD*
P1 Δ*lapD*	811	840
	wild type + p*srrA*	*lapC190* + p*srrA*
P1 Δ*srrA*	794	736

**Table 4:** MarA-mediated multicopy suppression of *lapC190* bacteria requires the presence of Mla system.

Recipient	P1 Donor					
	30 °C			43 °C		
	Δ*mlaC*	Δ*mlaD*	Δ*mlaCD*	Δ*mlaC*	Δ*mlaD*	Δ*mlaCD*
wt BW25113	1260	1380	1429	1412	1274	980
*lapC190* (SR23583)	623	710	690	12	14	9
*lapC190* + p*marA* (SR23660)	654	740	565	9	12	8

**Table 5:** The *srrA*-mediated multicopy suppression of *lapC190* bacteria requires the presence of cardiolipin synthase A encoded by the *clsA* gene.

Recipient	P1 Donor Δ*clsA*	
	30 °C	43 °C
wt	840	759
*lapC190* + vector alone	3	-
*lapC190* + p*srrA*^+^ with IPTG	331 small colonies	38

**Table 6:** Bacterial strains and plasmids used in this study.

Strains	Genotype	Reference
BW25113	*lacI*^q^ *rrnB*_T14_ Δ*lacZ*_WJ16_ *hsdR514* Δ*araBAD*_AH33_ Δ*rhaBAD*_LD78_	[70]
GK1942	BW25113+pKD46	[19]
W3110	λ^−^, *IN* (*rrnD-rrnE*)*1, rph-1*	CGSC, Yale
GK6075	BW25113 *lapC190*<>*cat*	[6]
SR23529	GK6075 *lapC190*<>*frt*	This study
SR23583	BW25113 *lapC190*<>*aph*	This study
SR23679	BW25113 *lapD*<>*aph*	[28]
SR23743	SR23679 *lapD<>frt*	[28]
SR9073	BW25113 *mlaC*<>*aph*	[42]
SR9078	BW25113 *mlaD*<>*aph*	[42]
SR23627	BW25113 *mlaCD*<>*aph*	This study
SR23630	GK6075 *mlaCD*<>*aph*	This study
SR19957	BW25113 *srrA*<>*aph*	[36]
SR23605	BW25113 *marA*<>*aph*	This study
SR7092	BW25113 *clsA*<>*aph*	Our collection
SR23320	BW25113 *clsA*<>*frt*	[28]
SR20066	BW25113 *dksA*<>*aph*	[36]
SR24224	BW25113 *pldA*<>*aph*	This study
GK1275	W3110 *lpxM*<>*aph*	[13]
GK1077	W3110 *lpxL*<>*aph*	[13]
SR23941	GK6075 *lpxL*<>*aph msbA* L412P	This study
SR23946	GK6075 *lpxM*<>*aph msbA* L412P	This study
SR19761	BW25113 + pCA24N	This study
SR23951	SR23529 + p*srrA*^+^	This study
SR23952	SR23951 *clsA*::Tn*10*	This study
SR23992	SR23529 + p*yfgM*^+^	This study
SR23994	SR23529 + p*dksA*^+^	This study
SR23996	SR23529 + p*artJ*^+^	This study
SR23998	SR23529 + p*acpP*^+^	This study
SR24000	SR23529 + p*accB*^+^	This study
SR24027	SR23529 + p*lapD*^+^	This study
SR24097	SR23529 + p*pldA*^+^	This study
SR23660	SR23591 + p*marA*^+^	This study
SR24099	SR23529 + p*marA*^+^ *mlaC*<>*aph*	This study
SR24100	SR23529 + p*marA*^+^ *mlaC*::Tn*10*	This study
SR24101	SR23529 + p*gnsA*^+^	This study
SR24103	SR23529 + p*yceJ*^+^	This study
SR24105	SR23529 + p*acpT*^+^	This study
SR24107	SR23529 + p*ymgG*^+^	This study
GK3592	BW25113 *sfhC21 zad220*::Tn*10* Δ*ftsH3*::Kan	[19]
SR24021	SR23529 + p*dksA*^+^	This study
SR24011	SR23529 + p*dksA* D74N	This study
SR24013	SR23529 + p*dksA* F82Y	This study
SR24015	SR23529 + p*dksA* S83A	This study
SR24017	SR23529 + p*dksA* L84A	This study
SR24019	SR23529 + p*dksA* E85A	This study
SR24056	SR23529 + p*lapD* ΔN20	This study
SR24058	SR23529 + p*lapD* (Δ100-132)	This study
SR24167	SR23529 + p*lapD* D69A Y70A R71A	This study
SR24170	SR23529 + p*lapD* L84A L85A P86A	This study
SR24173	SR23529 + p*lapD* N93A P94A F95A	This study
**Plasmids**	**Genotype**	**Reference**
pCA24N	IPTG-inducible expression vector cm^R^	[33]
pDUET	Expression vector	Our collection
pKD3	*oriR6K_g_*, *bla*(Amp^R^), *kan*, *rgnB*(Ter), *cat*	[70]
pKD13	*oriR6K_g_*, *bla*(Amp^R^), *kan*, *rgnB*(Ter)	[70]
pKD46	*araBp*-*gam*-*bet*-*exo*, *bla*(Amp^R^), *repA101*(ts)	[70]
pCP20	ts replicon with inducible FLP recombinase	[70]
pSR23599	*lapD*^+^ in pDUET	This study
pSR22189	*dksA*^+^ in pBR322 *lacI*^q^ tet^S^ amp^R^	[36]
pSR22505	*dksA*^+^ D74N in pBR322 *lacI*^q^ tet^S^ amp^R^	[36]
pSR22511	*dksA*^+^ F82Y in pBR322 *lacI*^q^ tet^S^ amp^R^	[36]
pSR22519	*dksA*^+^ S83A in pBR322 *lacI*^q^ tet^S^ amp^R^	[36]
pSR22498	*dksA*^+^ L84A in pBR322 *lacI*^q^ tet^S^ amp^R^	[36]
pSR22525	*dksA*^+^ E85A in pBR322 *lacI*^q^ tet^S^ amp^R^	[36]
JW0141	*dksA*^+^ in pCA24N cm^R^	[33]
JW0844	*artJ*^+^ in pCA24N cm^R^	[33]
JW0976	*gnsA*^+^ in pCA24N cm^R^	[33]
JW1044	*yceJ*^+^ in pCA24N cm^R^	[33]
JW1080	*acpP^+^* in pCA24N cm^R^	[33]
JW2497	*yfgM*^+^ in pCA24N cm^R^	[33]
JW3223	*accB*^+^ in pCA24N cm^R^	[33]
JW3440	*acpT*^+^ in pCA24N cm^R^	[33]
JW3794	*pldA*^+^ in pCA24N cm^R^	[33]
JW5178	*ymgG*^+^ in pCA24N cm^R^	[33]
JW5249	*marA*^+^ in pCA24N cm^R^	[33]
JW5539	*lapD*^+^ in pCA24N cm^R^	[33]
pSR14857	*srrA*^+^ in pCA24N cm^R^	This study
pEB797	pBAD-CBP-ACP (S36T)	[71]

## Data Availability

Data are contained within the article.
